# Evaluation of the immunogenicity of vaccine candidates developed using a baculovirus surface display system for Crimean-Congo hemorrhagic fever virus in mice

**DOI:** 10.3389/fmicb.2023.1107874

**Published:** 2023-03-16

**Authors:** Gang Zhang, Pu Wang, Lingling Jiang, Sheng Wang, Sinong Zhang, Yong Li

**Affiliations:** ^1^Key Laboratory of Ministry of Education for Conservation and Utilization of Special Biological Resources in Western China, Ningxia University, Yinchuan, China; ^2^School of Life Sciences, Ningxia University, Yinchuan, China

**Keywords:** Crimean-Congo hemorrhagic fever, Baculovirus expression systems, surface display, vaccine, immunological evaluation

## Abstract

Crimean-Congo hemorrhagic fever (CCHF), which has a fatality rate of 20–30%, is widely prevalent in several regions in Asia, Europe, and Africa and has spread to a wider range of areas in recent years. At present, there is a lack of safe and effective vaccines for the prevention of CCHF. In this study, we prepared three vaccine candidates, rvAc-Gn, rvAc-Np, and rvAc-Gn-Np, that encoded the CCHF virus (CCHFV) glycoprotein Gn and the nucleocapsid protein (Np) on the surface of baculovirus using an insect baculovirus vector expression system (BVES) and evaluated their immunogenicity in BALB/c mice. The experimental results showed that both CCHFV Gn and Np were expressed by the respective recombinant baculoviruses and anchored to the viral envelope. BALB/c mice were immunized, and all three recombinant baculoviruses showed significant humoral immunity. At the cellular level, the level of immunity in the rvAc-Gn group was significantly higher than that in the rvAc-Np and rvAc-Gn-Np groups, and the rvAc-Gn-Np coexpression group exhibited the lowest level of cellular immunity. In conclusion, the strategy of coexpressing Gn and Np in the baculovirus surface display system did not result in improvements in immunogenicity, whereas the recombinant baculovirus displaying Gn alone could induce significant humoral and cellular immunity in mice, indicating that rvAc-Gn has potential as a CCHF vaccine candidate. This study thus provides new ideas for the development of a CCHF baculovirus vaccine.

## 1. Introduction

Crimean-Congo hemorrhagic fever (CCHF) is a zoonotic disease that is transmitted to humans mainly by ticks, herbivorous domestic animals and pets (Negredo et al., 2017). Infection in animals only involves viraemia, whereas in humans, the clinical symptoms are fever, diarrhea, fatigue, and drowsiness. In severe cases, patients develop kidney lesions, liver failure, and lung damage and exhibit a mortality rate of approximately 30% (Garrison et al., 2019). There is no specific vaccine to prevent the disease. In 2019, the World Health Organization announced that 3 billion people worldwide were at risk of CCHF infection (World Health Organizations HEPaR, 2019). CCHF poses a widespread threat to public health due to its potential prevalence, high mortality, nosocomial infections, and difficulty in treatment and prevention (Belobo et al., 2021). A safe and effective vaccine is the most powerful measure for preventing CCHF virus (CCHFV) infection. Human immunization can reduce the number of CCHF cases, and animal immunization can reduce the risk of zoonotic transmission.

CCHFV consists of three negative-stranded RNA fragments: L, M, and S. The L fragment encodes an RNA-dependent RNA polymerase, the M fragment encodes the glycoproteins Gn and Gc on the viral envelope, and the S fragment encodes the nucleocapsid protein (Np; Zivcec et al., 2016; Garrison et al., 2019). Among these, the Gn and Gc glycoproteins contain neutralizing antigenic determinants that play important roles in virus absorption and invasion into host cells by inducing the body to produce neutralizing antibodies (Mohamed et al., 2021). The specific neutralizing antibodies induced by the Gc antigen subunit vaccine do not sufficiently protect mice from CCHFV infection, whereas the antibodies induced by the Gn antigen subunit vaccine protect mice from CCHFV infection *via* passive immunization (Ahmed et al., 2005). Moreover, the S fragment is relatively conserved and encodes an intracellular Np that more robustly activates T cells in response to Np antigenic epitopes compared with glycoproteins (Goedhals et al., 2017), and the Np can modulate intrinsic immunity by regulating proinflammatory and apoptotic pathways. Considering the characteristics of Gn and Np in CCHFV, we further investigated their immunogenicity.

This study used an established baculovirus expression vector system (BEVS), which has strong adjuvant activity and is safe and simple to prepare, for vaccine development (Martínez-Solís et al., 2019). This system has been applied to several commercial vaccines, such as the FDA-approved bivalent human papillomavirus vaccine Cervarix®, which was produced using BEVS and placed on the market in 2009 (Deschuyteneer et al., 2010), and a classical swine fever virus (CSFV) vaccine that was approved by the European Health Authorities in 2000 and entered the commercial phase (Pijlman et al., 2004). In addition, immunogenicity was enhanced by displaying the target gene on the surface of the baculovirus compared with that obtained with the expression of Np alone. The humoral and cellular immunity of the baculovirus recombinant proteins were evaluated in mice to provide new ideas for the development of CCHF vaccine candidates.

## 2. Materials and methods

### 2.1. Cells and viruses

Alfalfa silvery night moth (Sf9) cells (Invitrogen, United States) were cultured in Sf-900 II serum-free medium (SFM; Gibco, Grand Island, NY, United States) at 27°C. The baculovirus expression vector pFastBac Dual (Invitrogen, United States) was a gift from Prof. Yulong He of Zhejiang Sci-Tech University. The wild-type baculovirus rvA-dual was kept in our laboratory, and vector validation sequencing was performed by Jilin Kumi Biological Co.

### 2.2. Construction of the recombinant baculovirus surface display vector

Based on previous studies and the epidemiological investigation of CCHF in the Xinjiang region conducted by our laboratory (Kong et al., 2022a), the glycoproteins Gn (MN832721.2) and Np (MN832721.1) of the Chinese Xinjiang strain HANM18 (MN832721) were selected (Kong et al., 2022b), and the surface display sequence SPgp64 (GenBank AFO10080.1), the fluorescent proteins mCherry and eGFP and the histidine tag were cloned into the baculovirus pFastBac Dual vector to form the recombinant plasmids SPgp64-Gn-mCherry-TMDgp64, SPgp64-Np-eGFP-TMDgp64 and PH (SPgp64-Gn-mCherry-TMDgp64)-P10 (SPgp64-Np-eGFP-TMDgp64), which were synthesized by Anhui General Biotechnology Co.

### 2.3. Acquisition of recombinant bacmids

The constructed recombinant plasmids were transformed into DH10 Bac receptor cells (Biomed, China), and the recombinant bacmid was extracted using a kit (Beyotime, China) and verified by PCR amplification using M13 universal primers (F′: CCCAGTCACGACGTTGTAAAACG, R’: AGCGGATAACAATTTCACACAGG). Subsequently, Sf9 cells were transfected with the following mixture according to the instructions: 4 μL of transfection reagent, 1 μg of bacmid and Sf9 medium to a total volume of 100 mL. The P0 generation of the virus was then obtained after constant incubation.

### 2.4. Detection by direct fluorescence, indirect immunofluorescence and immunocolloidal gold electron microscopy

To observe the expression and localization of Gn protein and Np, Sf9 cells were observed 72 h, 96 h and 120 h after transfection by inverted fluorescence microscopy, and the localization of Gn protein and Np in Sf9 cells was observed by indirect immunofluorescence. The rvAc-Gn, rvAc-Np and rvAc-Gn-Np recombinant baculoviruses were added dropwise to cultured cells in 6-well plates at multiplicity of infection (MOI) of 0.1; the primary antibody was a mouse anti-His monoclonal antibody (1:500 dilution; Incubate for 8 h; Abcam), and the secondary antibodies were CoraLite 488-labeled goat anti-mouse IgG and CoraLite 594-labeled goat anti-mouse IgG (1:500 dilution; Incubate for 1 h; Proteintech). Protein fluorescence was observed at different wavelengths using a laser confocal microscope (Leica TCS SPE, Wetzlar, Germany). Viral supernatants were purified by sucrose density gradient centrifugation, and the recombinant baculoviruses were collected for immunoelectron microscopy with colloidal gold particles using the following primary antibodies: mouse anti-mCherry monoclonal antibody (1:1000 dilution; Abmart) and mouse anti-eGFP monoclonal antibody (preserved for this experiment). The secondary antibody protein-A-Gold (1:100 dilution; Bios) and electron microscopy grids were stained with 3% phosphotungstic acid and photographed using a transmission electron microscope at 80 kV (Hitachi H-7650, Tokyo, Japan).

### 2.5. Western blot analysis of recombinant baculovirus

Fluorescent tags may affect the immunogenicity of target proteins; thus, we sequenced the recombinant plasmids with the fluorescent tags removed, verified the bacmid sequences by PCR amplification, transfected Sf9 cells and passaged them to P1, P2 and P3 generations with an MOI of 0.5. Viral supernatants were collected, and the baculovirus recombinant proteins rvAc-Gn, rvAc-Np and rvAc- Gn-Np were identified by Western blotting; the primary antibody used was a mouse anti-His monoclonal antibody, and the secondary antibodies were CoraLite 488-labeled goat anti-mouse IgG and CoraLite 594-labeled goat anti-rabbit IgG (1:500 dilution; Proteintech).

### 2.6. Immunization experiments in mice

Thirty-five 7-week-old BALB/c mice (purchased from Beijing Viton Lever Laboratory Animal Technology Co., Ltd., weighing 16–20 g, Certificate of Conformity No. SCXK Jing2021-0006) were randomly divided into five groups (*n* = 9). Serum samples were collected from the posterior orbital sinus at 0 days, 14 days, 28 days, 35 days, and 42 days (Table 1). Three mice from each group were randomly selected and sacrificed at 35 days and 42 days by cervical dislocation, and their spleens were used for splenic lymphocyte value-adding experiments. The mouse experiments were performed according to the guidelines of the Animal Experimentation Ethics Committee of Ningxia Medical University.

**Table 1:** Mouse immunization strategy.

	Group	Immunization time (DAI)	Immunization dose
1	PBS	0, 14, 28	100 μL
2	rvAc-dual	0, 14, 28	10^7^ PFU
3	rvAc-Gn	0, 14, 28	10^7^ PFU
4	rvAc-Np	0, 14, 28	10^7^ PFU
5	rvAc-Np-Gn	0, 14, 28	10^7^ PFU

### 2.7. Serum IgG analysis in mice

Serum antibody levels were measured by indirect enzyme-linked immunosorbent assay (ELISA), and the experimental procedure was previously described in detail. The antigens were Gn protein and Np and were prepared using a prokaryotic expression system at a concentration of 0.1 mg/mL. The primary antibodies were serum samples analyzed on 0 days, 14 days, 28 days, 35 days, and 42 days after the first immunization (diluted 1:100 with the blocking solution). The secondary antibodies were labeled goat anti-mouse IgG (1:1000 dilution, Proteintech). After termination of the reaction, the absorbance values at 450 nm were measured using an enzyme marker (Bio-Tek, Winooski, VT, United States), and all ELISA experiments were performed in triplicate.

### 2.8. Splenic lymphocyte proliferation analysis

The experimental procedure was performed as described previously (Xu et al., 2012) based on stimulation with 2 μg/mL Gn and Np and using RPMI-1640 (Gibco) medium as a negative control, and 5 mg/mL cutin A as a positive control. Three replicates of each sample were prepared and incubated at 37°C for 42 h. MTT solution (5 mg/mL, 20 μl, Sigma) was added to each well, and the absorbance values were measured at 490 nm using an enzyme marker (Bio-Tek, Winooski, VT, United States). The stimulation index was calculated for each group as follows: stimulation index (SI) = OD490 nm of antigen-stimulated cells (Gn and Np)/mean OD490 nm of negative control (RPMI-1640).

### 2.9. Statistical analysis

The statistical analyses were performed using GraphPad Prism® V version 6 for Windows (GraphPad Software, San Diego, CA, United States). The data are expressed as the means ± standard deviations. The Bonferroni *post hoc* test was used to compare the immune response between the groups. The data were significantly different if *p* < 0.05.

## 3. Results

### 3.1. CCHFV Gn and np recombinant baculovirus vector construction and identification

**Figure 1:**
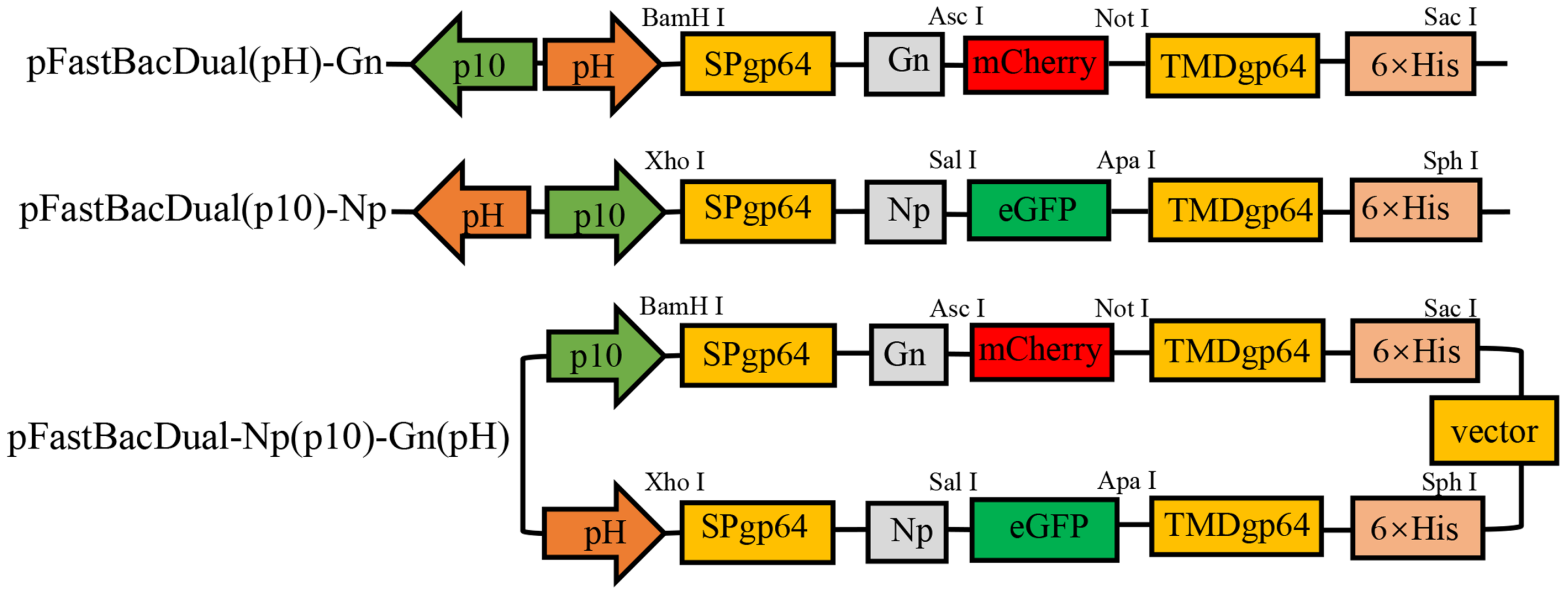
Recombinant plasmid mapping.

**Figure 2:**
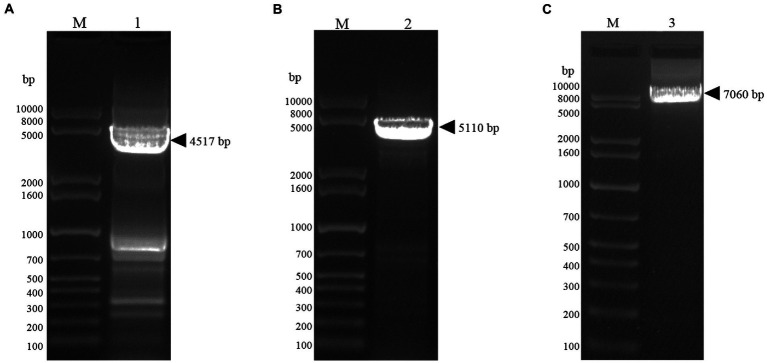
Bacmid PCR validation. **(A)** Lane1, Gel electrophoresis image of bacmid-Gn-mCherry identified by PCR. **(B)** Lane2, Gel electrophoresis image of bacmid-Np-eGFP PCR identified by PCR. **(C)** Lane3, Gel electrophoresis image of bacmid-Gn-mCherry-Np-eGFP PCR identified by PCR.

In this study, the plasmids pFastBac Dual (pH)-Gn, pFastBac Dual (p10)-Np and pFastBac Dual-Gn (pH)-Np (p10) were constructed (Figure 1). After gene synthesis, the correctly sequenced recombinant plasmids were transfected into *E*. *coli* DH10Bac cells, and the obtained recombinant bacmids were amplified using M13 universal primers. Verification of the obtained recombinant bacmids was completed by PCR amplification using M13 universal primers. Bacmid-Gn-mCherry (4,517 bp), Bacmid-Np-eGFP (5,110 bp), and Bacmid-GnmCherry-Np-eGFP (7,060 bp), and the electrophoretic band positions were consistent with the expected sizes (Figure 2).

### 3.2. Antigen expression of recombinant baculovirus rvAc-Gn-mCherry, rvAc-np-eGFP and RVAC-Gn-mCherry-NP-EGFP in cells

**Figure 3:**
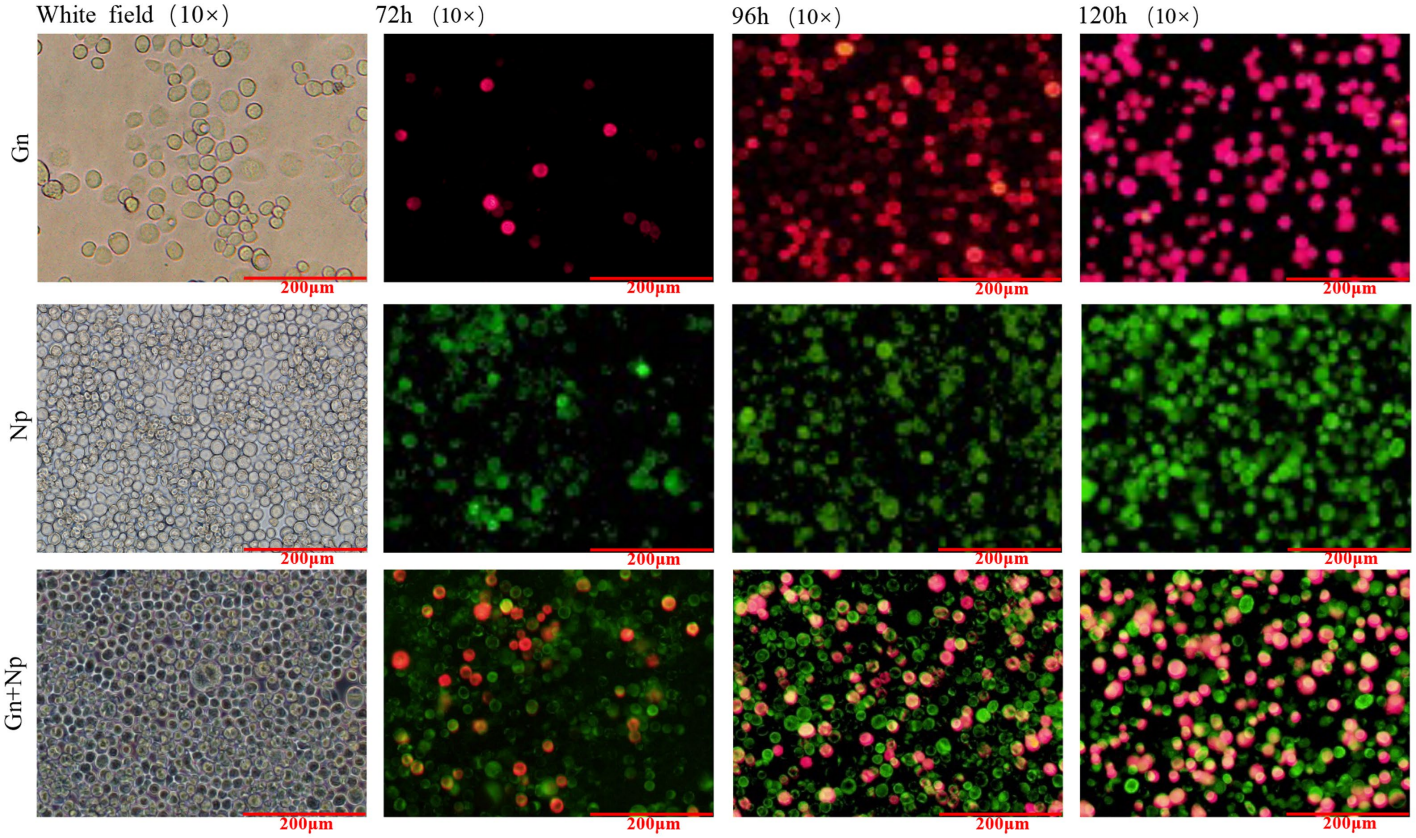
Direct fluorescence observation of rvAc-Gn-mCherry, rvAc-Np-eGFP and rvAc-Gn-mCherry-Np-eGFP recombinant bacmids transfected into Sf9 cells (72 h, 96 h, and 120 h).

**Figure 4:**
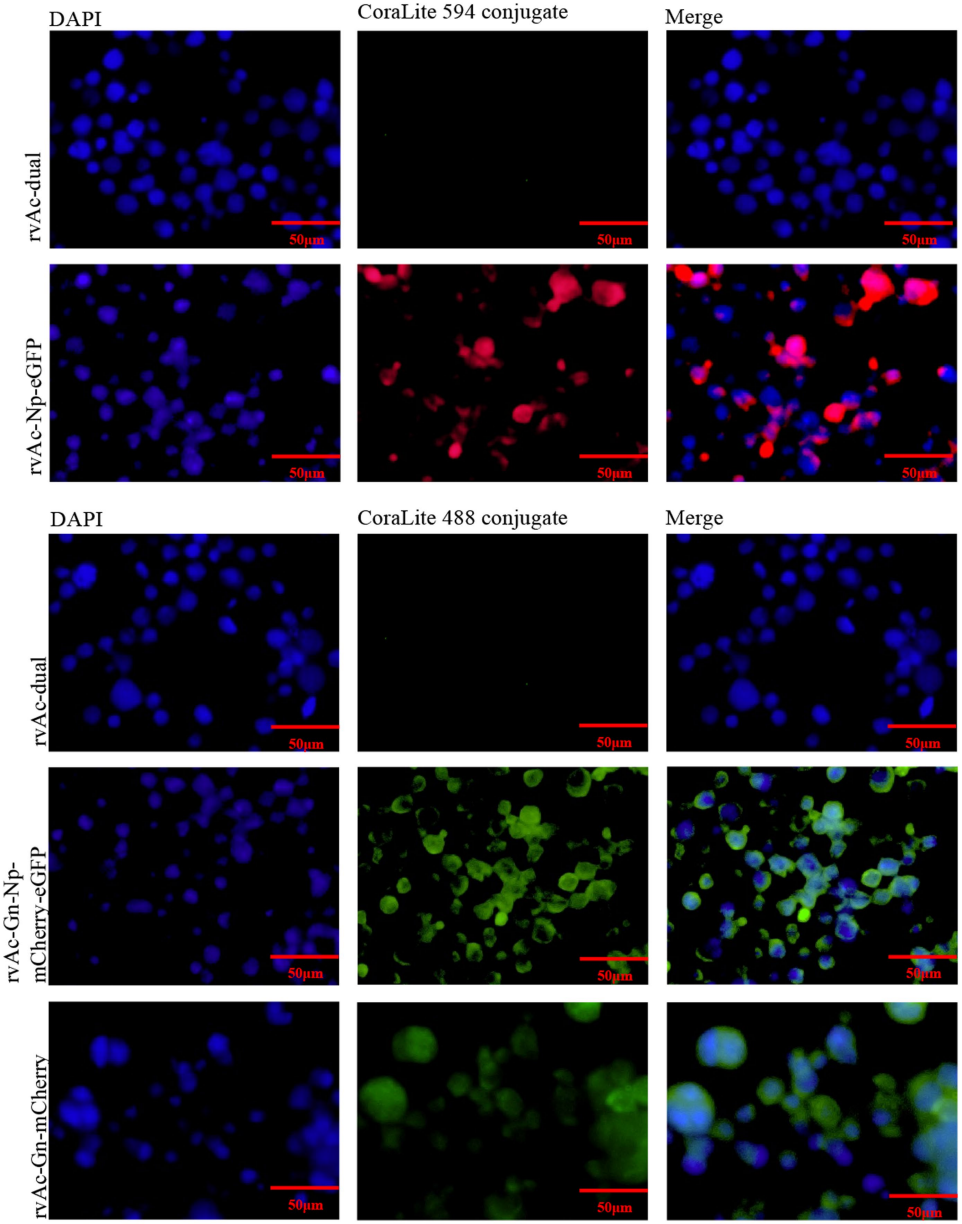
Indirect immunofluorescence of recombinant baculovirus- and wild-type virus-infected cells.

Sf9 cells were infected with the recombinant baculoviruses rvAc-Gn-mCherry, rvAc-Np-eGFP and rvAc-Gn-mCherry-Np-eGFP and observed under different excitation (white, green and blue) wavelengths after incubation at 27°C for 72 h, 96 h, and 120 h. All three recombinant baculoviruses expressed fluorescent proteins (Figure 3). We further examined the localization of Gn protein and Np in the cells by indirect immunofluorescence and laser confocal microscopy, and the target proteins expressed by rvAc-Np-eGFP, rvAc-Gn-mCherry and rvAc-Gn-mCherry-Np-eGFP were localized near the cell membrane (Figure 4).

**Figure 5:**
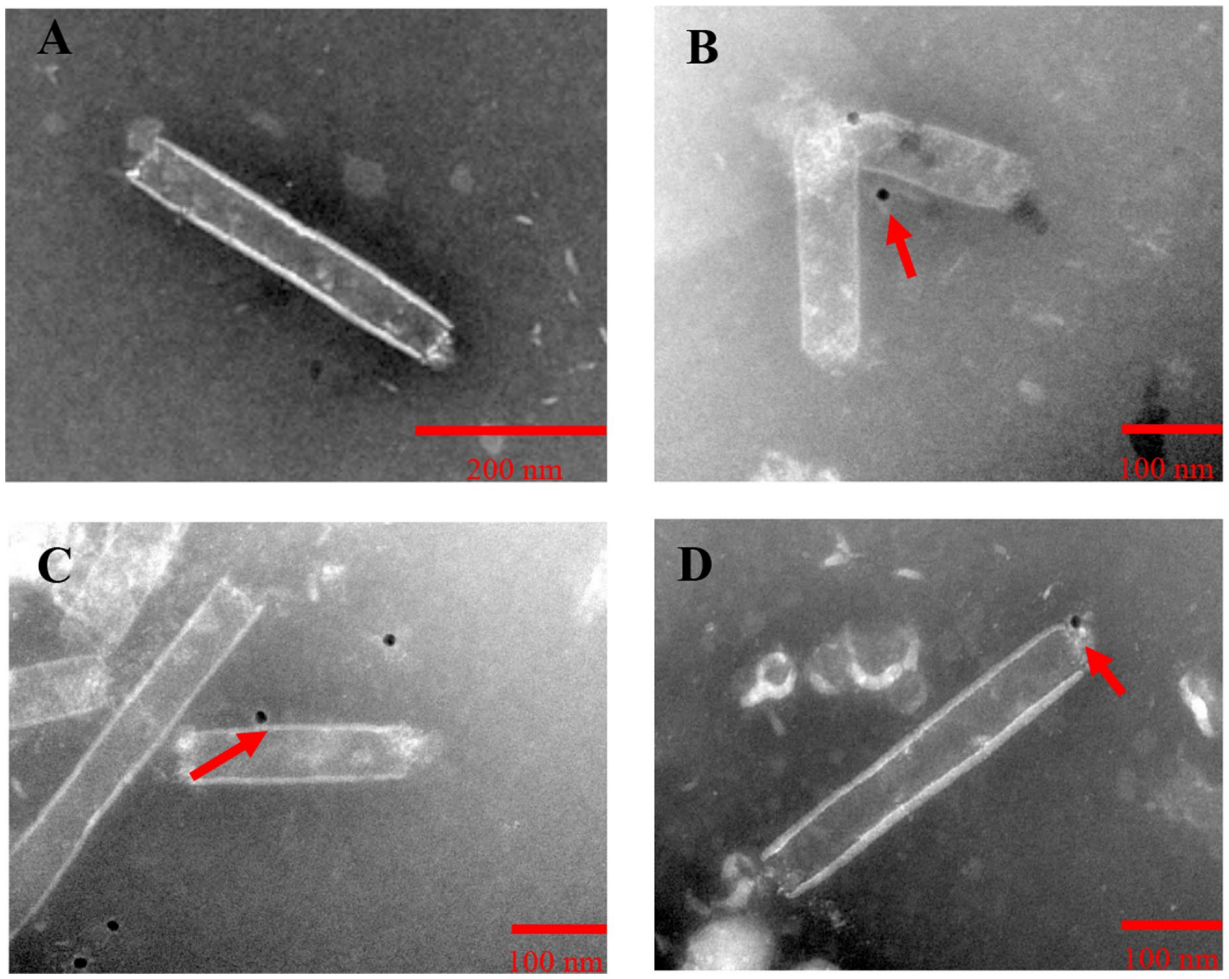
Immunoelectron microscopic observation of purified recombinant baculoviruses. **(A)** rvAc-dual was incubated with mCherry monoclonal antibodies. **(B)** rvAc-Gn-mCherry was incubated with mCherry monoclonal antibodies. **(C)** rvAc-Np-eGFP was incubated with eGFP monoclonal antibodies. **(D)** rvAc-Gn-mCherry-Np-eGFP was incubated with eGFP monoclonal antibodies. On the surface of the recombinant baculovirus incubated with mCherry and eGFP monoclonal antibodies, colloidal gold particles with a diameter of approximately 10 nm aggregated in the vesicle region, whereas no colloidal gold particles were found on the surface of wild-type baculovirus.

To examine the expression of Gn protein and Np on baculoviruses, the four recombinant baculoviruses rvAc-dual, rvAc-Gn-mCherry, rvAc-Np-eGFP and rvAc-Gn-mCherry-Np-eGFP were observed by immunoelectron microscopy with colloidal gold particles. The results showed that all the recombinant viruses had colloidal gold particles with a diameter of approximately 10 nm aggregated on the capsid region, indicating that the fluorescent proteins carrying Gn and Np were anchored on the surface of recombinant baculoviruses (Figure 5).

### 3.3. Analysis of humoral and cellular immunity in mice

**Figure 6:**
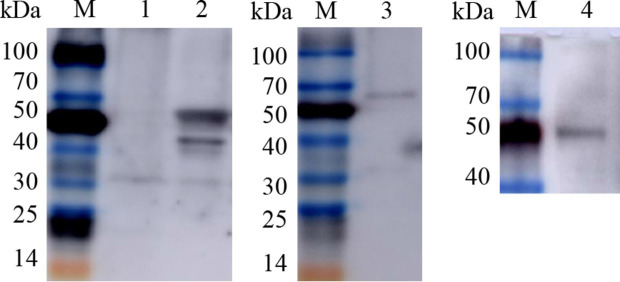
Western blot identification of recombinant baculovirus surface display particles. M, TransGen, Blue Plus Protein Marker; Lanes 1, rvAc-dual; Lane 2, Western blot (68.6 kDa/45.7 kDa); Lane 3, rvAc-Np (68.6 kDa); Lane 4, rvAc-Gn (45.7 kDa).

**Figure 7:**
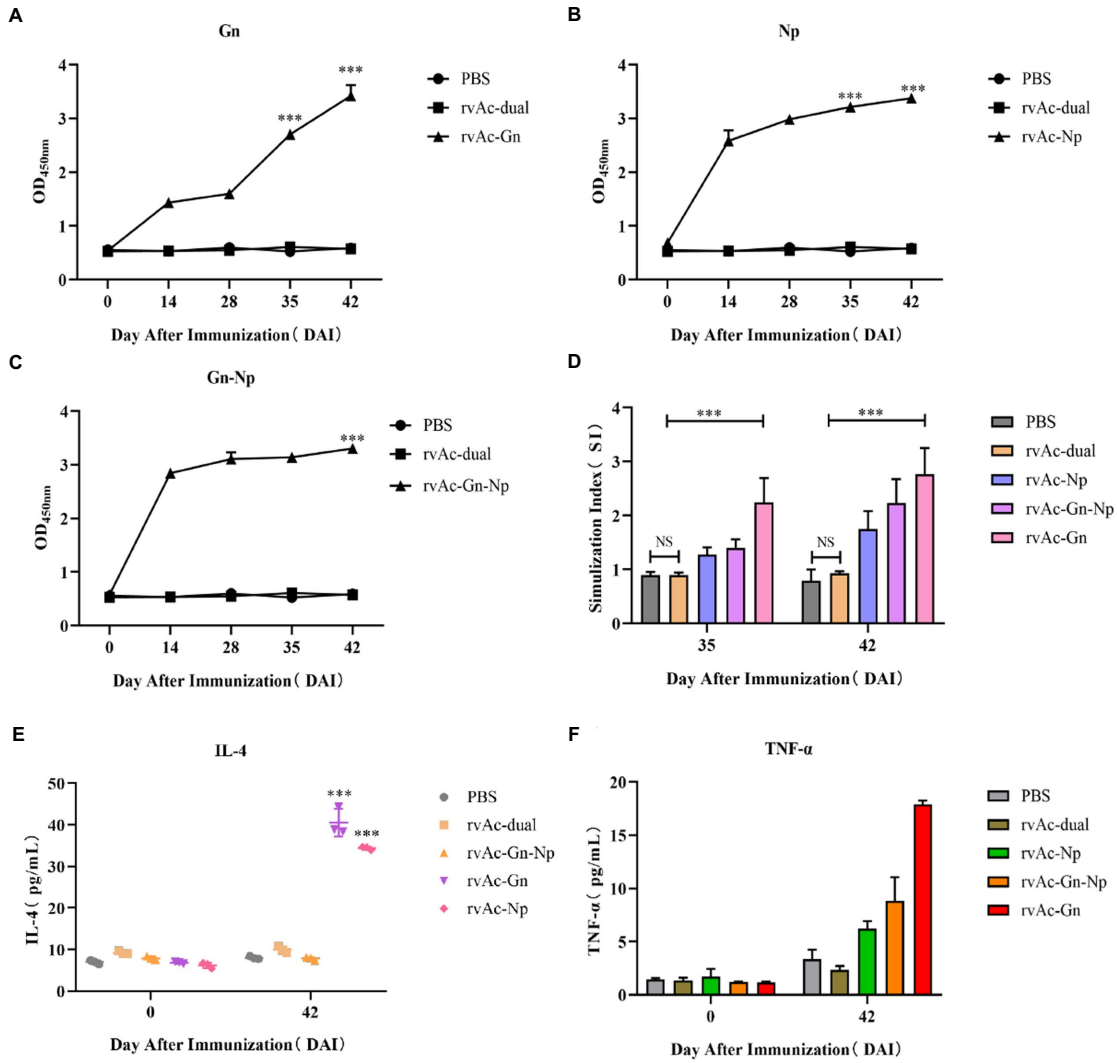
Immunogenicity analysis of mice. The immunogenicity of four groups of recombinant baculovirus-infected mice was evaluated by measuring the serum IgG levels **(A–C)**. **(A)** Anti-Gn protein IgG levels. **(B)** Anti-Np IgG levels. **(C)** Anti-Gn-Np IgG levels. The X-axis indicates the number of days the serum samples were collected after immunization, and the Y-axis indicates the mean OD450 of the serum samples. **(D)** For the splenic lymphocyte stimulation assay, the data in the figure represent the stimulation indices of splenic lymphocyte samples collected at 35 and 42 DAI, and the assay was repeated three times for all the samples. The data are presented as the means + variances (*N* = 4). NS, no significant difference; ***p < 0.001, significant difference (Bonferroni assay). To further evaluate the cellular immune responses of the baculovirus-infected mice, the serum IL-4 **(E)** and TNF-α **(F)** levels were measured. The data in the graph represent the total serum levels of the cytokines in the samples collected at 0 days and 42 days. The assay was repeated three times for all the samples, and the error bars represent the standard deviations (SDs). ***p < 0.001, highly significant difference compared with the rvAc-dual PBS group (Bonferroni assay).

The three constructed recombinant vectors were modified by removing the fluorescent labeling to obtain rvAc-Gn, rvAc-Np and rvAc-Gn-Np, and Western blot assays showed that the Gn and Np antigens were correctly expressed (Figure 6). To investigate the effects of recombinant baculovirus rvAc-Gn, rvAc-Np and rvAc-Gn-Np antigens on humoral immunity in mice, the immunoglobulin G (IgG) levels in mouse serum were measured by indirect ELISA. The rvAc-Gn group had significantly higher IgG levels than the negative control group, PBS group and wild-type virus group at 35 days (Figure 7A), and these levels peaked at 42 days (*p < 0*.*001*). In the rvAc-Np group, the total IgG levels in serum were significantly higher (*p < 0*.*001*) at 14 days after immunization and peaked at 42 days (Figure 7B), and the differences in the total IgG levels among the rvAc-Gn, rvAc-Np and rvAc-Gn-Np groups were not significant (7A-C; *p < 0*.*001*). To investigate the cellular immune response to recombinant baculovirus in mice, we examined the stimulation index (SI) of mouse splenic lymphocytes at 35 days and 42 days (Figure 7C). The results showed that the SIs of the rvAc-Gn, rvAc-Np and rvAc-Gn-Np groups were higher than that of the control group, but the SI of the rvAc-Gn group was significantly higher than that of the other two groups at 35 days and 42 days (*p <* 0.001; Figure 7D).

To further evaluate cellular immune responses, we examined the changes in the IL-4 and TNF-α levels in mouse serum (Figures 7E,F). The results showed that the serum levels of IL-4 and TNF-α were significantly higher in the mice belonging to the rvAc-Gn and rvAc-Np groups, and the IL-4 level of the rvAc-Gn-Np group was not different from that of the negative control group. The TNF-α level of the rvAc-Gn group was significantly higher than that of the rvAc-Np group (*p <* 0.001). The rvAc-Gn, rvAc-Np and rvAc-Gn-Np groups had TNF-α levels of 17.887 ± 0.379 pg/mL, 6.211 ± 0.693 pg/mL and 8.804 ± 2.260 pg/mL, respectively, and IL-4 levels of 40.530 ± 3.288 pg/mL, 34.394 ± 0.387 pg/mL and 16.509 ± 1.530 pg/mL, respectively.

## 4. Discussion

In recent years, ecological improvements and migratory birds have led to the spread of tick-borne CCHF to a wider area, and CCHF thus poses a public safety threat to many regions (Spengler et al., 2019; Portillo et al., 2021). In addition to the development of specific effective therapeutic drugs, vaccines are an important measure for preventing this disease. Vaccines against CCHFV, such as DNA vaccines (Sahin et al., 2021), subunit vaccines (Wang et al., 2022), virus-like particle vaccines (Hinkula et al., 2017) and plant vector vaccines (Ghiasi et al., 2011), have some immunogenicity but are also defective, and their effectiveness cannot be demonstrated due to the lack of reasonable animal models and the limited access to higher-level BL4 laboratories. There is no internationally recognized, safe and effective vaccine for CCHF (Sana et al., 2022). Therefore, the development of a vaccine for CCHF is a topic of great importance.

In the present study, we developed three vaccine candidates encoding CCHFV Gn protein and Np on the surface of baculovirus and evaluated their immunogenicity in BALB/c mice. Gn and Gc glycoproteins are important components of the viral capsid and have been widely reported to be immunogens for CCHF vaccines (Wu et al., 2017; Tipih and Burt, 2020). In SW-13 cell neutralization assays, Gn antigen-based vaccines prevented CCHFV infection, but during passive immunization, only some Gc antigen-based vaccines protected mice from CCHFV infection, which suggests that the neutralization of CCHFV infection may depend on factors other than the characteristics of the antibodies (Bertolotti-Ciarlet et al., 2005). In addition, the formation and role of the Np in ribonucleoprotein complexes is essential for viral replication, and this protein is a major antigen for inducing immune responses to many bunyaviruses (Dowall et al., 2017) and has 91–99% amino acid homology among Np fragments of different strains (Ghiasi et al., 2011); thus, vaccines developed using the Np as an immunogen have broad protective power (Goedhals et al., 2017). Although the Np does not induce neutralizing antibody production, the induced production of cytotoxic T lymphocytes remains detectable several years after infection, suggesting that Np could also be a potential target antigen for CCHF vaccines. In this study, we prepared rvAc-Gn, rvAc-Np and rvAc-Gn-Np baculoviruses that expressed the target protein in Sf9 cells, and the Gn protein and Np were anchored to the baculovirus capsid surface by the signal peptide SPgp64 (Figure 5), as described previously (Abbink et al., 2017).

After the mice were immunized, the IgG antibody levels were significantly higher in the rvAc-Gn, rvAc-Np and rvAc-Gn-Np groups than in the control group, indicating that all three viral vector vaccine candidates could induce significant humoral immune responses in mice (*p* < 0.001). In the splenic lymphocyte stimulation assay, the SI of the rvAc-Gn group was significantly higher than those of the other two groups (*p* < 0.001), and the highest SI was detected 42 days after immunization, indicating that rvAc-Gn induced stronger cellular immunity than rvAc-Np in mice. Other researchers have obtained similar results in studies of CCHFV Gn and Np vaccines (Bertolotti-Ciarlet et al., 2005; Karaaslan et al., 2021).

T-cell immunity is associated with the clearance of organismal pathogens in patients (Lombe et al., 2021), and in CCHF survivors, CD4+ effector cells are predominantly the Th1 type, which secrete TNF-α to activate neutrophils to phagocytose and digest pathogens and promote cellular immune responses (Jin et al., 2004), whereas Th2-type cells secrete IL-4 to induce specific antibody production (Vancott et al., 1996). After three doses of rvAc-Gn, rvAc-Np, and rvAc-Gn-Np, the levels of Th1- and Th2-type cytokines were elevated in mice; in the rvAc-Gn group, the serum IL-4 levels were 40.53 pg/mL, and the serum TNF-α levels were 17.887 pg/mL, which indicated significant Th1- and Th2-type cellular immune responses (*p* < 0.001). There is a dynamic balance between the Th1-type cellular immune response, which plays a protective role in the body, and the Th2-type cellular immune response, which helps the body clear the infection. rvAc-Gn-Np did not induce significant Th1 and Th2 cellular immunity but mainly triggered humoral immune responses. Vaccine candidates with strong immunogenicity that provide total protection from the virus in animals (Li et al., 2016) may be related to the nature of CCHFV itself. Moreover, immunogenicity could not be assessed in comparison with commercial vaccines due to the lack of a reasonable commercial vaccine, and pathogenic experiments could not be performed to assess its efficacy as a vaccine candidate due to lack of access to a higher-rated BL4 laboratory. However, it is clear that the immunogenicity of Gn is significantly higher than that of Np, and rvAc-Gn has the potential to be a vaccine candidate against CCHFV infection.

## 5. Conclusion

In conclusion, we constructed three vaccine candidates (rvAc-Gn, rvAc-Np and rvAc-Gn-Np) using an insect baculovirus vector expression system (BEVS) and evaluated their immunogenicity in mice. The results showed that the Gn and Np coexpression candidate vaccine rvAc-Gn-Np has limited immunogenicity. In contrast, the rvAc-Gn vaccine candidate exhibits strong cellular and humoral immunity and has the potential to be a vaccine candidate for treating CCHFV infection.

## Data availability statement

The original contributions presented in the study are included in the article/Supplementary material, further inquiries can be directed to the corresponding authors.

## Ethics statement

The animal study was reviewed and approved by Ningxia Medical University.

## Author contributions

GZ carried out then experimental work and wrote the manuscript. PW crunched the numbers. LJ conducted the gene design and culture of Sf9 cells. SW and SZ provided technical guidance. YL provided the initial idea, designed the study, and revised the manuscript. All authors contributed to the manuscript changes and read and approved the submitted version.

## Funding

This work was supported by the National Natural Science Foundation of China (grant number: 32130104) and the Key Research and Development Program of Ningxia Hui Autonomous Region (grant number: 2021BEF02028).

## Conflict of interest

The authors declare that the research was conducted in the absence of any commercial or financial relationships that could be construed as a potential conflict of interest.

## Publisher’s note

All claims expressed in this article are solely those of the authors and do not necessarily represent those of their affiliated organizations, or those of the publisher, the editors and the reviewers. Any product that may be evaluated in this article, or claim that may be made by its manufacturer, is not guaranteed or endorsed by the publisher.
